# A Case Report of the Atypical Presentation of Multiple Myeloma Manifesting as a Sternal Mass

**DOI:** 10.7759/cureus.65004

**Published:** 2024-07-20

**Authors:** Himanshi Kaushik, Nishtha Manuja, Kanchan D Devde, Amol Dongre

**Affiliations:** 1 Department of Medical Oncology, Jawaharlal Nehru Medical College, Datta Meghe Institute of Medical Sciences (Deemed to be University), Wardha, IND; 2 Department of Medicine, Jawaharlal Nehru Medical College, Datta Meghe Institute of Medical Sciences (Deemed to be University), Wardha, IND

**Keywords:** hypercalcemia, crab, plasmacytoma, plasma cell, bone marrow examination

## Abstract

The diagnosis of multiple myeloma (MM) is made based on the presence of either marrow clonal plasma cells > 10% or an extramedullary or bony plasmacytoma confirmed by biopsy. Additionally, at least one of the SLiM (sixty years, light chain ratio, magnetic resonance imaging)-CRAB (calcium elevation, renal insufficiency, anemia, and bone lesions) myeloma-defining events must be present. MM typically presents with symptoms such as fatigue due to anemia, kidney failure, hypercalcemia, and bone pain. It is uncommon, though, for MM to manifest as a single bone mass. We report the case of a 65-year-old male who did not fit the criteria for solitary bone plasmacytoma and presented with an unusual sternal tumor. The patient was diagnosed with MM despite not having any of the traditional symptoms such as low back pain, weight loss, anemia, or hypercalcemia. The diagnosis was based on a bone marrow examination, which showed 50% plasma cells. Radiation therapy and systemic chemotherapy were then used to treat him. The patient's symptoms, radiological findings, and biopsy results are described in detail, emphasizing the difficulty and intricacy of correctly diagnosing this uncommon manifestation of MM. This case highlights the need for a comprehensive and multidisciplinary strategy to diagnose and treat atypical presentations of MM, making sure that all possible diagnostic pathways are investigated in order to achieve accurate and timely diagnosis and treatment.

## Introduction

The disease known as multiple myeloma (MM) starts in the plasma cells, a subset of white blood cells essential for generating antibodies to combat infections in the body. In MM, abnormal plasma cells gather and proliferate; this proliferation can lead to various complications, including bone damage, anemia, kidney dysfunction, and increased susceptibility to infections. With a median diagnostic age of roughly 69 years, MM usually affects people in the age group of 65-74 years [1,2]. It commonly presents with fatigue due to anemia, kidney failure, hypercalcemia, and bone pain [3,4]. Atypical presentations of MM like hyperviscosity syndrome, multiple cystic chest swellings, and pleural effusion have been reported but sternal involvement as the primary presenting symptom is infrequently documented in the medical literature [5-7].

In this case report, we provide a comprehensive overview of the clinical, histopathological, and radiological aspects, highlighting the diagnostic challenges encountered. We detail the patient's symptoms, imaging findings, and biopsy results, illustrating the complexity of accurately diagnosing this unusual presentation of MM. This case underscores the importance of a thorough and multidisciplinary approach in the diagnosis and management of atypical presentations of MM.

## Case presentation

**Figure 1:**
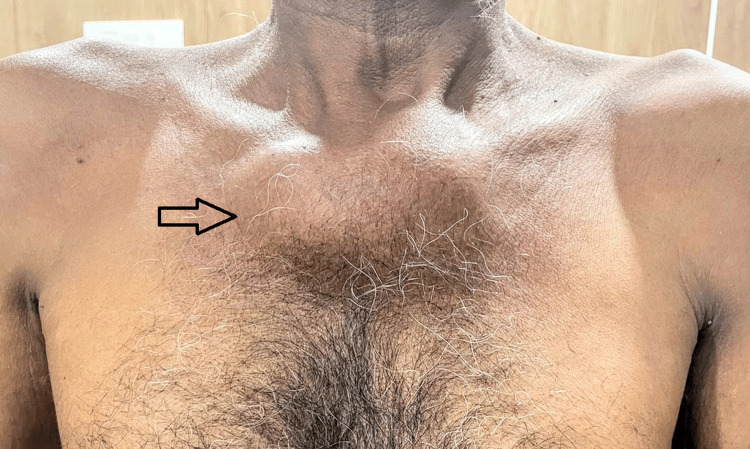
Swelling (black arrow) in the sternal region

A 65-year-old male without any concomitant conditions showed up with a sternal wall enlargement that had been steadily growing over the previous six months and was sometimes accompanied by minor pain. On physical examination, a fixed non-tender mass of around 10 x 9 cm with hard consistency, well-defined margins, and smooth surface was palpated over the sternum (Figure 1).

Laboratory investigations like blood routine, alkaline phosphatase, lactate dehydrogenase (LDH), kidney function, and calcium were normal (Table 1).

**Table 1:** Blood investigations

Blood Investigations	Results	Reference Range
Hemoglobin (g/dL)	14.8	12.0-17.5
White Blood Cell (10^3^U/L)	9.9	4.5-11.0
Platelets (10^5^/ul)	2.29	1.5-4.5
Mean Corpuscular Volume (fl)	100.1	80-100
Creatinine (mg/dL)	0.8	0.61-1.24
Calcium (mg/dl)	8	8.5-10.5
Alkaline Phosphatase (IU/L)	150	44-147
Total Protein (g/dL)	7.2	6-8
Lactate Dehydrogenase (U/L)	500	140-280
Albumin (g/dL)	3.8	3.5–5.0

**Figure 2:**
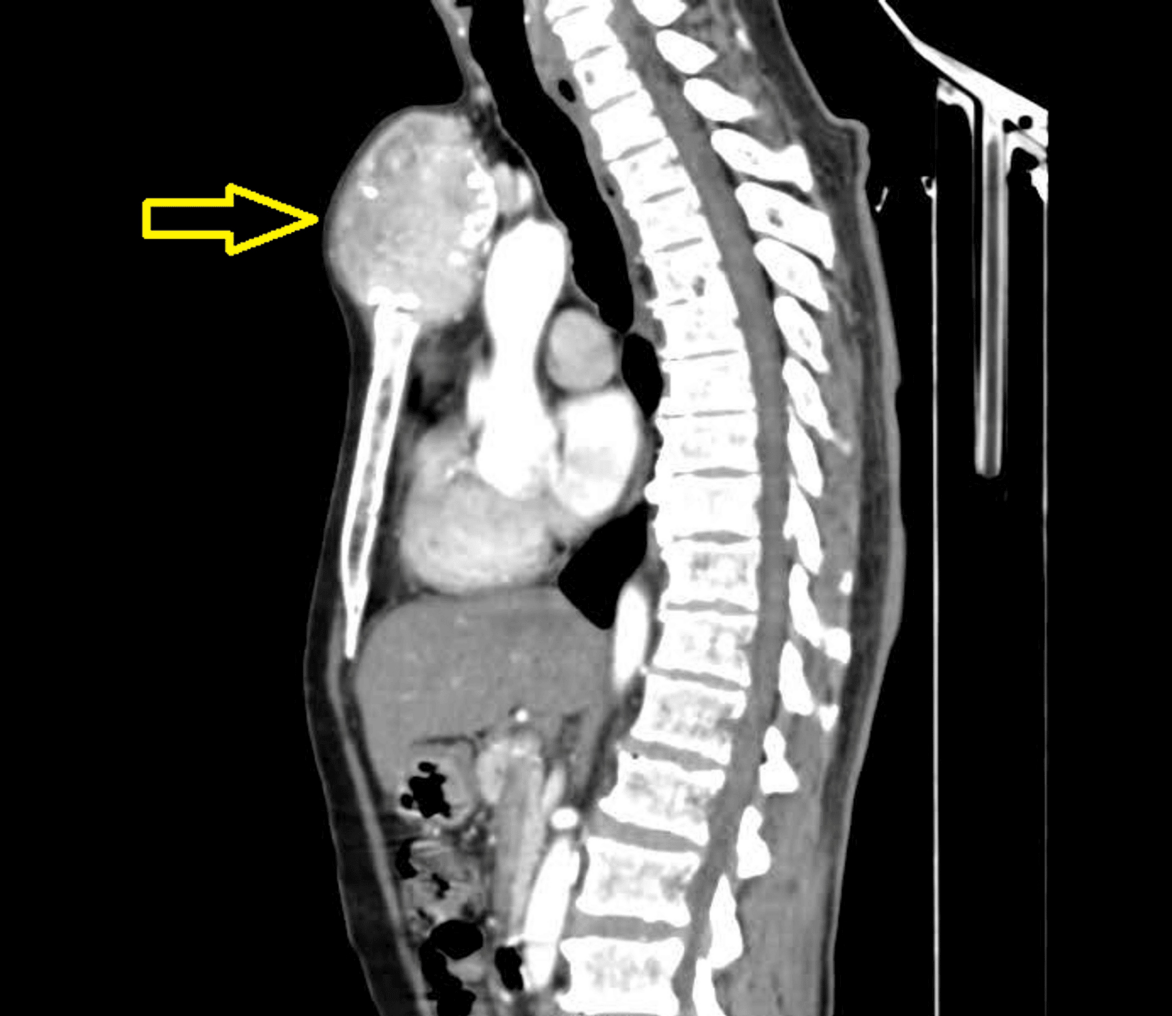
CT scan showing mass lesion (yellow arrow) in manubrium sterni

Computed tomography (CT) of the chest suggested an ill-defined soft tissue expansile lytic lesion in the manubrium sternum measuring 9.9 x 7.8 x 6.6 cm (Figure 2). The sternal mass fine needle aspiration cytology (FNAC) was done and cytopathology revealed lesions of plasma cell pathology of MM. A skeletal evaluation revealed no lytic lesions. The skull X-ray came out normal. No signs of anemia, hypercalcemia, or renal involvement indicative of systemic myeloma were observed. Serum protein electrophoresis (SPEP) results showed no evidence of monoclonal gammopathy, as there was no M spike in the gamma globulin region. Notably, serum β2 microglobulin levels were within the normal range of 0-3 ug/mL. The kappa light chain, however, was noticeably higher at 4167.17 mg/L. In addition, the kappa/lambda ratio, which was 101.79, was much higher than the 0.26-1.65 typical reference range.

**Figure 3:**
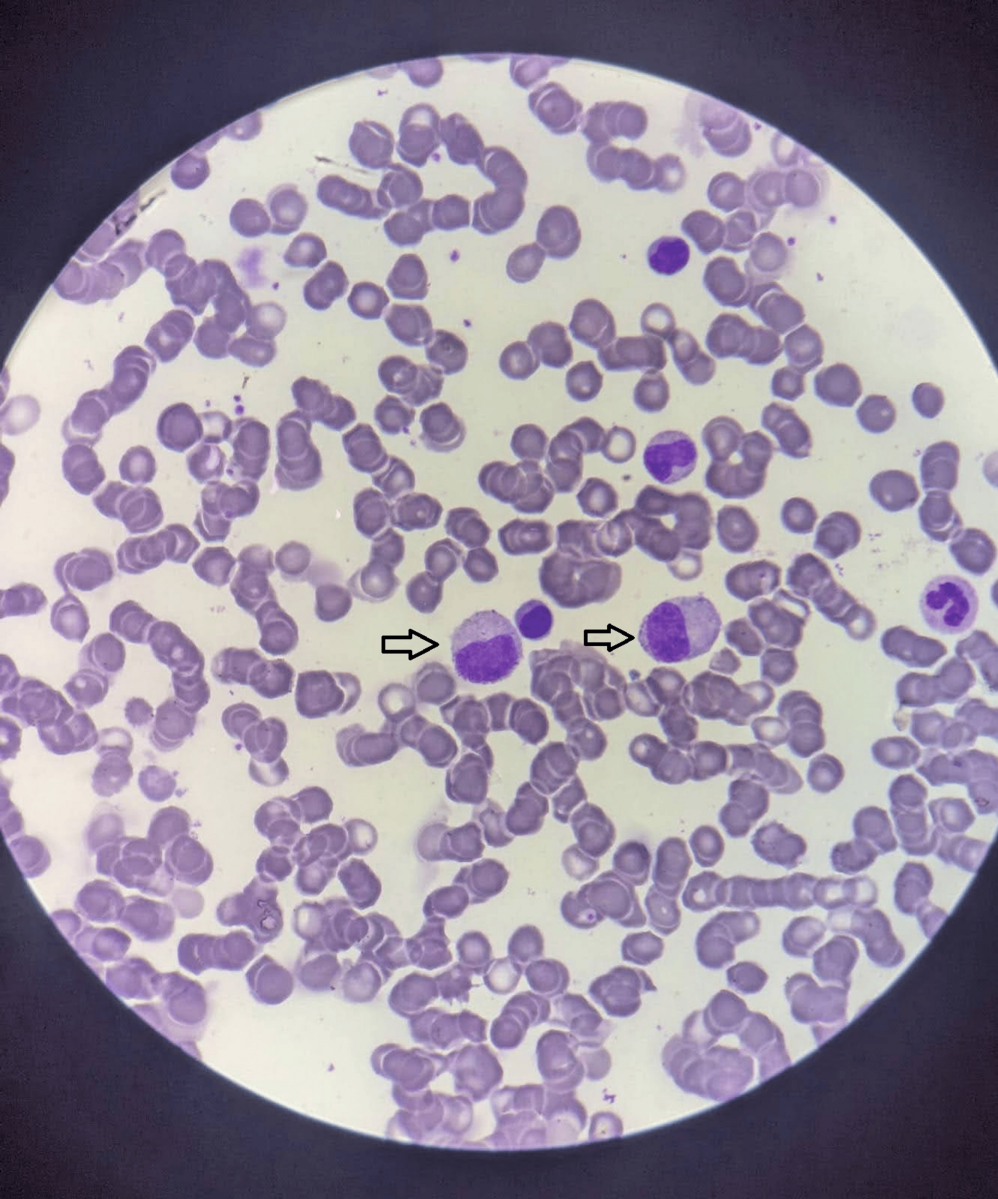
Plasma cell (black arrows) on bone marrow aspiration

Figure 3 shows the results of a bone marrow examination that showed plasma cell dyscrasia, with 50% of plasma cells present, which is indicative of MM. The patient was assigned a stage III MM according to the Revised International Staging System (R-ISS). 

A diagnosis of atypical presentation MM was established based on the clinical presentation, imaging results, and biopsy data. The Velcade, Revlimid, dexamethasone (VRD) regimen, comprising bortezomib, dexamethasone, and, lenalidomide, was initiated for the patient. Additionally, radiation therapy was planned to target the sternal mass to address the localized tumor burden and alleviate symptoms.

## Discussion

MM makes up only 1% of all cancers among hematological malignancies, which is a relatively small fraction [3]. MM's clinical significance lies in its potential to cause a range of systemic complications, including bone lesions, renal impairment, and immunodeficiency [4]. 

MM is now defined by the International Myeloma Working Group (IMWG) using biomarkers in addition to the classic CRAB (calcium elevation, renal insufficiency, anemia, and bone lesions) characteristics [8-10]. Two requirements must be satisfied to diagnose MM: either biopsy-verified extramedullary or bony plasmacytoma, or bone marrow clonal plasma cells ≥ 10%. Furthermore, at least one of the SLiM(Sixty years, light chain ratio, MRI)-CRAB myeloma-defining events [4].

Raised calcium levels (above 11.5 mg/dL), renal insufficiency (creatinine > 2 mg/dL or creatinine clearance < 40 mL/min), anemia (hemoglobin < 10 g/dL or 2 g/dL below the lower limit of normal), and the presence of bone lesions found on imaging studies (MRI, X-ray, etc.) are all considered part of the CRAB criteria. These standards, which represent the systemic effects of the illness on different organ systems, are applied in the diagnosis and severity evaluation of MM [8]. According to the IMWG, the presence of bone disease in MM is confirmed by the identification of one or more osteolytic lesions using skeletal radiography, whole-body MRI, or whole-body fluorine-2-fluoro-2-deoxy-d-glucose (FDG) positron emission tomography/computerized tomography (PET/CT). This explanation guarantees that different imaging modalities can successfully contribute to diagnosing and monitoring bone involvement in the disease. Additionally, MM-defining biomarkers, also known as SLiM characteristics (60 years, light chain ratio, MRI), have been identified by IMWG. These consist of one or more of the following: the presence of multiple focal marrow lesions (non-osteolytic) on MRI; a free light chain ratio (FLC) of 100 or higher with the involved free light chain equal to or greater than 100 mg/L; or clonal plasma cells equal to or greater than 60% in the bone marrow [8]. These incidents collectively are referred to as SLiM-CRAB [11,12].

A common presentation of solitary plasmacytoma is a solitary lesion with evidence of clonal plasma cells that is verified by biopsy in both soft tissue and bone. But, to classify it as a solitary plasmacytoma, three further requirements need to be met: no clonal plasma cells in the normal bone marrow, normal results on MRI and skeletal survey, and no end-organ damage, as demonstrated by the absence of the CRAB criterion [13]. The standard of care for solitary bone plasmacytoma (SBP) is radiotherapy [3,14]. Although a set protocol does not exist, radiotherapy doses usually consist of 40-50 Gy given over four weeks [8,11,12,15]. Less than 10% of bone marrow plasma cells are clonal in solitary plasmacytomas with little marrow involvement. In contrast, the patient in our case report had 50% clonal bone marrow plasma cells, which resulted in the diagnosis of MM with an atypical presentation. To differentiate it from a single plasmacytoma, a higher percentage denotes a more widespread infiltration of aberrant plasma cells in the bone marrow.

In this case report, a biopsy-confirmed single mass with clonal plasma cells presented a highly atypical appearance for systemic MM, indicating a diagnosis more in line with solitary plasmacytoma. The patient in question did not exhibit any of the usual symptoms associated with MM, including low back pain, weight loss, anemia, or hypercalcemia symptoms. Additionally, the patient had normal levels of erythrocyte sedimentation rate (ESR) and serum calcium, with no renal dysfunction. SPEP did not show an M band. However, upon evaluation, bone marrow examination revealed 50% plasma cells, indicating a more extensive involvement suggestive of MM. According to the treatment guidelines for systemic MM, chemotherapy is the preferred therapeutic approach. Its presentation as a single bone mass is rare and should be managed accordingly. Treatment needs to be modified, as does the role of radiotherapy in such types of cases.

## Conclusions

This case underscores the importance of comprehensive diagnostic evaluations in patients with atypical presentations of plasma cell disorders. Initially, a diagnosis of solitary plasmacytoma was considered due to the presence of a single bone mass and the absence of the typical signs and symptoms of MM, such as low back pain, weight loss, anemia, and hypercalcemia. Additionally, the patient did not meet the SLiM-CRAB criteria commonly used to diagnose MM. However, the discovery of extensive bone marrow involvement ultimately confirmed a diagnosis of systemic MM. The management of such cases should adapt standard MM treatment protocols to address the unique presentation, balancing chemotherapy and radiotherapy as appropriate. According to treatment guidelines for systemic MM, chemotherapy is the preferred therapeutic approach. However, the rare presentation of MM as a single bone mass necessitates a tailored treatment strategy. Radiotherapy, typically a primary treatment for solitary plasmacytoma, must be reconsidered within the broader context of systemic MM management. This case highlights the need for individualized treatment plans to effectively manage atypical presentations of systemic MM.
